# Demographic Characteristics of Persons Vaccinated During the First Month of the COVID-19 Vaccination Program — United States, December 14, 2020–January 14, 2021

**DOI:** 10.15585/mmwr.mm7005e1

**Published:** 2021-02-05

**Authors:** Elizabeth M. Painter, Emily N. Ussery, Anita Patel, Michelle M. Hughes, Elizabeth R. Zell, Danielle L. Moulia, Lynn Gibbs Scharf, Michael Lynch, Matthew D. Ritchey, Robin L. Toblin, Bhavini Patel Murthy, LaTreace Q. Harris, Annemarie Wasley, Dale A. Rose, Amanda Cohn, Nancy E. Messonnier

**Affiliations:** ^1^CDC COVID-19 Response Team; ^2^Stat-Epi Associates, Inc., West Palm Beach, Florida; ^3^General Dynamics Information Technology, Falls Church, Virginia.

In December 2020, two COVID-19 vaccines (Pfizer-BioNTech and Moderna) were authorized for emergency use in the United States for the prevention of coronavirus disease 2019 (COVID-19).* Because of limited initial vaccine supply, the Advisory Committee on Immunization Practices (ACIP) prioritized vaccination of health care personnel^†^ and residents and staff members of long-term care facilities (LTCF) during the first phase of the U.S. COVID-19 vaccination program (*1*). Both vaccines require 2 doses to complete the series. Data on vaccines administered during December 14, 2020–January 14, 2021, and reported to CDC by January 26, 2021, were analyzed to describe demographic characteristics, including sex, age, and race/ethnicity, of persons who received ≥1 dose of COVID-19 vaccine (i.e., initiated vaccination). During this period, 12,928,749 persons in the United States in 64 jurisdictions and five federal entities^§^ initiated COVID-19 vaccination. Data on sex were reported for 97.0%, age for 99.9%, and race/ethnicity for 51.9% of vaccine recipients. Among persons who received the first vaccine dose and had reported demographic data, 63.0% were women, 55.0% were aged ≥50 years, and 60.4% were non-Hispanic White (White). More complete reporting of race and ethnicity data at the provider and jurisdictional levels is critical to ensure rapid detection of and response to potential disparities in COVID-19 vaccination. As the U.S. COVID-19 vaccination program expands, public health officials should ensure that vaccine is administered efficiently and equitably within each successive vaccination priority category, especially among those at highest risk for infection and severe adverse health outcomes, many of whom are non-Hispanic Black (Black), non-Hispanic American Indian/Alaska Native (AI/AN), and Hispanic persons (*2*,*3*).

Data on COVID-19 vaccine doses administered in the United States are collected by vaccination providers and reported to CDC through multiple sources, including jurisdictions, pharmacies, and federal entities, who use various reporting methods including immunization information systems,^¶^ Vaccine Administration Management System,** and direct data submission. Data on first vaccine doses administered during December 14, 2020–January 14, 2021, and reported to CDC by January 26, 2021, were analyzed to describe demographic characteristics, including sex, age, and race/ethnicity among persons who received ≥1 dose of COVID-19 vaccine. Age was calculated based on date or year of birth and date of vaccine administration and was categorized as <18, 18–29, 30–39, 40–49, 50–64, 65–74, or ≥75 years. Race and ethnicity were combined and categorized as Hispanic/Latino, White, Black, non-Hispanic Asian (Asian), AI/AN, non-Hispanic Native Hawaiian or other Pacific Islander (NH/PI), non-Hispanic multiple/other,^††^ or unknown (if either race or ethnicity was reported as unknown^§§^ or not reported because of jurisdictional policy or law).^¶¶^ Analyses were conducted using SAS (version 9.4; SAS Institute).

During the first month of the U.S. COVID-19 vaccination program, 12,928,749 persons received at least 1 dose of COVID-19 vaccine (Figure). Vaccination was initiated by persons in all 64 jurisdictions and five federal entities reporting data to CDC. Among 12,537,841 (97.0%) vaccine recipients with reported sex, 63.0% were women and 37.0% were men (Table). Among 12,924,116 (99.9%) persons whose age was known, 55.0% were aged ≥50 years, 16.8% were aged 40–49 years, and 28.2.% were aged 18–39 years. Among 6,706,697 (51.9%) persons whose race/ethnicity was known, 60.4% were White and 39.6% represented racial and ethnic minorities, including 14.4% categorized as multiple or other race/ethnicity, 11.5% Hispanic/Latino, 6.0% Asian, 5.4% Black, 2.0% AI/AN, and 0.3% NH/PI. Race/ethnicity was unknown or not reported for 6,222,052 (48.1%) persons initiating vaccination. Across jurisdictions and federal entities, the percentage of persons initiating vaccination with race/ethnicity that was unknown or not reported ranged from 0.2% to 100% (median = 39.6%; interquartile range = 25.3%–66.1%).

**FIGURE:**
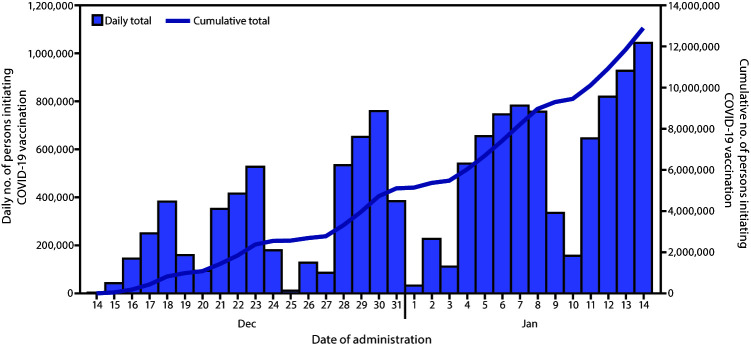
Number of persons initiating COVID-19 vaccination, by date of vaccine administration (N = 12,928,749) — United States, December 14, 2020–January 14, 2021* **Abbreviation:** COVID-19 = coronavirus disease 2019. * Vaccines administered December 14, 2020–January 14, 2021, and reported to CDC by January 26, 2021.

**TABLE:** Demographic characteristics of persons initiating COVID-19 vaccination — United States, December 14, 2020–January 14, 2021*

Characteristic (no. [%] with available information)	No. (%)^†^
**Overall**	**12,928,749 (100.0)**
**Sex (12,537,841 [97.0])**	
Male	4,639,073 (37.0)
Female	7,898,768 (63.0)
**Age group,^§^ yrs (12,924,116 [99.9])**	
<18	4,837 (<0.1)
18–29	1,433,086 (11.1)
30–39	2,207,222 (17.1)
40–49	2,175,305 (16.8)
50–64	3,350,610 (25.9)
65–74	1,732,522 (13.4)
≥75	2,020,534 (15.6)
**Race/Ethnicity**^¶^ **(6,706,697 [51.9])**	
White, non-Hispanic	4,047,795 (60.4)
Hispanic/Latino	773,858 (11.5)
Black, non-Hispanic	359,934 (5.4)
Asian, non-Hispanic	405,227 (6.0)
AI/AN, non-Hispanic	134,127 (2.0)
NH/PI, non-Hispanic	20,585 (0.3)
Multiple/Other, non-Hispanic**	965,171 (14.4)

**Abbreviations**: AI/AN = American Indian/Alaska Native; COVID-19 = coronavirus disease 2019; NH/PI = Native Hawaiian or Other Pacific Islander.

* Vaccines administered December 14, 2020–January 14, 2021, and reported to CDC by January 26, 2021.

**^†^** Percentages were calculated among persons with available demographic characteristics.

**^§^** Pfizer-BioNTech COVID-19 vaccine is authorized for persons aged ≥16 years, and Moderna COVID-19 vaccine is authorized for persons aged ≥18 years under Food and Drug Administration Emergency Use Authorizations. Ages that were outside of the expected range (<16 years or >120 years) were treated as unknown, which represented <0.1% of persons initiating vaccination.

^¶^ Race/ethnicity was not reported or was unknown for all persons initiating vaccination in six jurisdictions. The six jurisdictions not reporting race/ethnicity have a total population of approximately 18.9 million, which represents nearly 6% of the overall U.S. population.

** Represents persons identified as being non-Hispanic and having multiple race categories selected or being non-Hispanic and having “other race” selected.

## Discussion

During the first month of the U.S. COVID-19 vaccination program, 12,928,749 persons received ≥1 dose of COVID-19 vaccine, representing approximately 4% of the total U.S. population and 5% of the U.S. population aged ≥16 years.*** If vaccination was only provided to persons in the Phase 1a priority groups (health care personnel and LTCF residents), coverage among the 24 million persons included in these groups might have been as high as 50% (*1*). However, this is likely an overestimate because persons outside of the 1a priority group were vaccinated because of variation in implementation of national guidance at the jurisdictional and local levels (e.g., Florida and Texas expanded vaccination to all persons aged ≥65 years).^†††^

Among persons who received the first vaccine dose and had available data for the respective demographic characteristic variable, 63.0% were women, 55.0% were aged ≥50 years, and 60.4% were White, which likely reflects the demographic characteristics of the persons (health care personnel and LTCF residents) recommended to be vaccinated in the Phase 1a priority group (*4*,*5*). Data from the 2019 American Community Survey show that 60% of health care workers were White, 16% were Black, 13% were Hispanic, and 7% were Asian; however, race and ethnicity varied widely by occupation and setting (*6*). Women also account for approximately three fourths of persons employed in the health care industry (*7*). In addition, the 2015–2016 National Study of Long-Term Care Providers found that 65% of nursing home residents were women, 75% were White, 14% were Black, and 5% were Hispanic (*8*).

Interpretation of data from the analysis of COVID-19 vaccination initiation is limited by the high percentage of records with unknown or missing race/ethnicity information and the unknown proportions of priority groups (health care personnel versus LTCF residents) among early vaccine recipients. Differences in how race and ethnicity data are collected and categorized, for example 14.4% of persons initiating vaccination reported as multiple or other race/ethnicity, also make comparisons difficult. The percentage of persons initiating vaccination who were Black appears lower relative to the percentage of persons who are Black among health care personnel and LTCF residents. Overall, 39.6% of persons who were vaccinated represented racial and ethnic minorities. Because persons who are Black, AI/AN, or Hispanic have been found to have more severe outcomes from COVID-19 than persons who are White, careful monitoring of vaccination by race/ethnicity is critical (*2*,*9*).

The findings in this report are subject to at least three limitations. First, race/ethnicity was unknown for approximately one half of the population who initiated vaccination during the first month of the COVID-19 vaccination program in the United States. In addition, the proportion of persons with unknown race/ethnicity varied across jurisdictions, including six jurisdictions that reported no race/ethnicity data.^§§§^ In addition, a high proportion of persons receiving vaccination were categorized as non-Hispanic, multiple or other races, whereas the population estimates from the 2019 American Community Survey^¶¶¶^ 1-year population were 2.8% non-Hispanic, multiple or other races. Thus, the findings presented in this study might not be generalizable to all persons initiating COVID-19 vaccination in the United States. The large proportion of missing data also might result in biased estimates of race/ethnicity, particularly if some groups are more likely than others to have race/ethnicity reported as unknown. Second, vaccine administration data reported to CDC include limited data elements and did not allow for stratification by the prioritized populations (health care personnel and LTCF residents) in the initial phase of the vaccination campaign. Therefore, it was not possible to directly compare the observed demographic patterns among persons initiating vaccination to demographic characteristics of prioritized populations. Finally, implementation of the ACIP recommendations, including subprioritization, varied by jurisdiction, with some jurisdictions changing and expanding their priority populations during the first month of the vaccination program.

Although these data reflect characteristics of persons initiating vaccination during the initial phase of the U.S. COVID-19 vaccination program and have several limitations, the findings underscore the need for more complete reporting of race and ethnicity data at the provider and jurisdictional levels to ensure rapid detection of and response to potential disparities in COVID-19 vaccine administration. Jurisdictions should monitor the demographic characteristics of vaccinated persons to identify emerging disparities. In addition, as vaccination expands to include additional groups, monitoring coverage by the Social Vulnerability Index, which uses U.S. Census Bureau variables to identify communities that might need support, will be useful to ensure equity and to identify communities where focused immunization efforts might be required.**** CDC is working with jurisdictions to use these types of analyses to help direct efforts to bring vaccines to their communities and ensure that no persons are left behind. These data from the first month of the COVID-19 vaccination program indicate substantial progress in administration of the COVID-19 vaccine. To increase coverage among persons in Phase 1a, as vaccination expands into additional populations, unvaccinated health care personnel and LTCF residents should continue to be offered COVID-19 vaccine. Equitable and sustainable COVID-19 vaccine administration in all populations requires focus on groups with lower vaccine receipt who might face challenges with access or vaccine hesitancy.

**Summary**

**What is already known about this topic?**

In December 2020, two COVID-19 vaccines were authorized for emergency use in the United States. The first groups prioritized for vaccination included health care personnel and long-term care facility residents.

**What is added by this report?**

During the first month of the U.S. COVID-19 vaccination program, approximately 13,000,000 persons received ≥1 dose of vaccine. Among persons with demographic data, 63.0% were women, 55.0% were aged ≥50 years, and 60.4% were non-Hispanic White.

**What are the implications for public health practice?**

As the vaccination program expands, it is critical to ensure efficient and equitable administration to persons in each successive vaccine priority category, especially those at highest risk for infection and severe health outcomes.

## Footnotes

*
https://www.cdc.gov/vaccines/covid-19/eua/index.html

† Health care personnel include persons working in settings such as hospitals, long-term care facilities, outpatient clinics, public health clinics, emergency medical services, and pharmacies.

§ Sixty-four jurisdictions include 50 states, the District of Columbia, five cities (Chicago, Philadelphia, San Antonio, Houston, and New York City), and eight territories or freely associated states; five federal entities also received a direct allocation of vaccine (Federal Bureau of Prisons, U.S. Department of Defense, U.S. Department of State, Indian Health Services, and Veterans Health Administration) and report administration data to CDC.

¶
https://www.cdc.gov/vaccines/programs/iis/index.html

**
https://www.cdc.gov/vaccines/covid-19/reporting/vams/program-information.html

†† Represents persons identified as being non-Hispanic and having multiple race categories selected or non-Hispanic and “other race” selected.

§§ If ethnicity was identified as Hispanic and race was unknown, the person was classified as Hispanic.

¶¶ Race/ethnicity was not reported because of jurisdictional policy or law or was unknown for all persons initiating vaccination in six jurisdictions.

*** Population denominator data from U.S. Census Bureau 2019 American Community Survey 1-year population estimates. 2018 population estimates are used for American Samoa, Federated States of Micronesia, Guam, Marshall Islands, Northern Mariana Islands, Palau, and U.S. Virgin Islands.

††† http://www.floridahealth.gov/newsroom/2020/12/122920-2046-covid-vac.pr.html; https://dshs.texas.gov/news/releases/2020/20201221.aspx

§§§ The six jurisdictions not reporting race/ethnicity have a total population of approximately 18.9 million, which represents nearly 6% of the overall U.S. population.

¶¶¶
https://data.census.gov/cedsci/table?q=hispanic&tid=ACSDP1Y2019.DP05&hidePreview=false

**** The Social Vulnerability Index uses U.S. Census variables to help local officials identify communities that might need support as the result of a disaster. https://www.atsdr.cdc.gov/placeandhealth/svi/index.html
